# Hyperostosis Cranialis Interna Presenting With Cerebrospinal Fluid Leak: A Case Report and Review of Literature

**DOI:** 10.7759/cureus.78949

**Published:** 2025-02-13

**Authors:** Ahmed S Alsaleh, Abdullah I BinGhaith, Yazeed Alsuliman

**Affiliations:** 1 Otolaryngology, King Fahad Medical City, Riyadh, SAU; 2 College of Medicine, King Saud University, Riyadh, SAU; 3 Otolaryngology - Head and Neck Surgery, King Fahad Medical City, Riyadh, SAU

**Keywords:** csf leak, encephalocele, hyperostosis, secondary intracranial hypertension, skull base repair

## Abstract

This report is of a case of hyperostosis cranialis internal (HCI), a rare disease characterized by the abnormal thickening of cranial bones, usually in the frontal and parietal regions. It is an uncommon disease that has clinical significance because of its possible symptoms: neurologic, aesthetic concerns, or asymptomatic, which may incidentally be found on imaging. Detailed attention has been given to the case of a 32-year-old female patient who presented with a two-year history of right clear, salty nasal discharge, anosmia, and band-like headache. Computed tomography and magnetic resonance imaging findings helped in the diagnosis. The laboratory studies and treatment options have been described. A discussion on pathophysiology, differential diagnoses, and management is also done with the hope of adding to this limited literature on HCI. The implications for diagnosis and management in clinical practice are shown to be direct results of characterizing HCI as a unique bone disorder, whereby a better understanding of this condition is provided.

## Introduction

Hyperostosis cranialis interna (HCI) is a rare and poorly understood condition primarily affecting the internal surfaces of the cranial bones. It is distinct from other hyperostosis syndromes, such as hyperostosis frontalis internal (HFI), due to its diffuse involvement and potential systemic associations [1]. This condition is often discovered incidentally through imaging studies conducted for unrelated reasons, as symptoms may be subtle or entirely absent in the early stages [2].

Although its exact etiology is still unknown, it is generally recognized to be of autosomal dominant inheritance [3]. Other hypotheses propose its multifactorial origin, implicated by genetic predisposition, hormonal influence, and metabolic disorder, in which HFI is particularly included [4-6].

Among bone remodeling disorders, Paget’s disease of bone presents a notable differential diagnosis, characterized by abnormal bone turnover that leads to progressive skeletal deformities and complications. While it is one of the more common metabolic bone diseases in certain populations, its exact pathogenesis remains unclear, involving both genetic and environmental factors [7]. Similarly, osteopetrosis is a rare condition marked by increased bone density due to defective osteoclast function. It manifests in various forms, ranging from severe infantile cases with life-threatening complications to milder adult-onset types with a more benign course [8]. Additionally, osteitis fibrosa cystica, a skeletal manifestation of severe hyperparathyroidism, is characterized by excessive bone resorption leading to bone pain, cystic lesions, and fractures. Though now rare due to early detection of hyperparathyroidism, it remains a relevant differential diagnosis in cases of metabolic bone disease [9]. Another important consideration is fibrous dysplasia, a benign bone disorder caused by a postzygotic activating mutation in the *GNAS* gene, leading to abnormal fibro-osseous tissue development. It can present as a monostotic or polyostotic lesion, often involving the craniofacial bones, ribs, and long bones, sometimes associated with McCune-Albright syndrome [10].

The limited number of documented cases makes it difficult to establish definitive diagnostic criteria or therapeutic guidelines. This case report represents an in-depth analysis of a patient presenting with HCI, adding to the knowledge of its clinical spectrum, diagnostic challenges, and management approaches.

## Case presentation

A 32-year-old female patient, not known to have any medical illness, was referred to our rhinology unit with a two-year history of right clear, salty nasal discharge exacerbated by leaning forward, post nasal drip, anosmia, and band-like headache. There were no visual disturbances, pulsatile tinnitus, or history of nasal or neurosurgery, or trauma.

**Figure 1:**
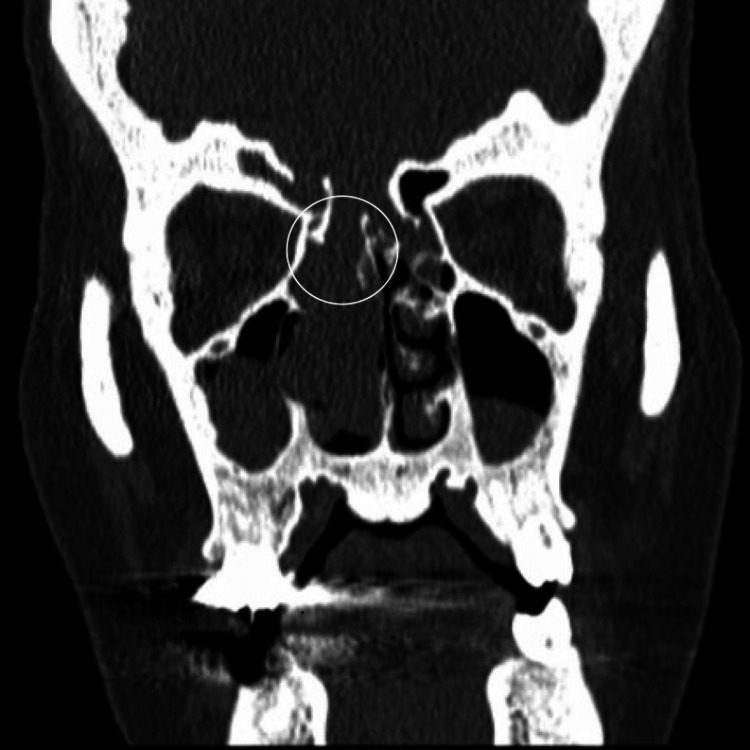
CT scan (coronal view) showing a skull base defect at the right cribriform plate (white circle) with encephalocele filling the right nasal cavity

Endoscopic examination showed a large encephalocele medial to the middle turbinate completely occluding the right nasal cavity. A CT scan of the sinuses, without contrast enhancement, revealed a bilateral diffuse polypoid mucosal thickening involving multiple sinuses, complete obliteration of the right posterior nasal passage, and severe stenosis of the ostiomeatal complex. Significantly, there was evidence of multiple bony erosions and defects at the right cribriform plate and frontal sinus roof (Figure 1).

**Figure 2:**
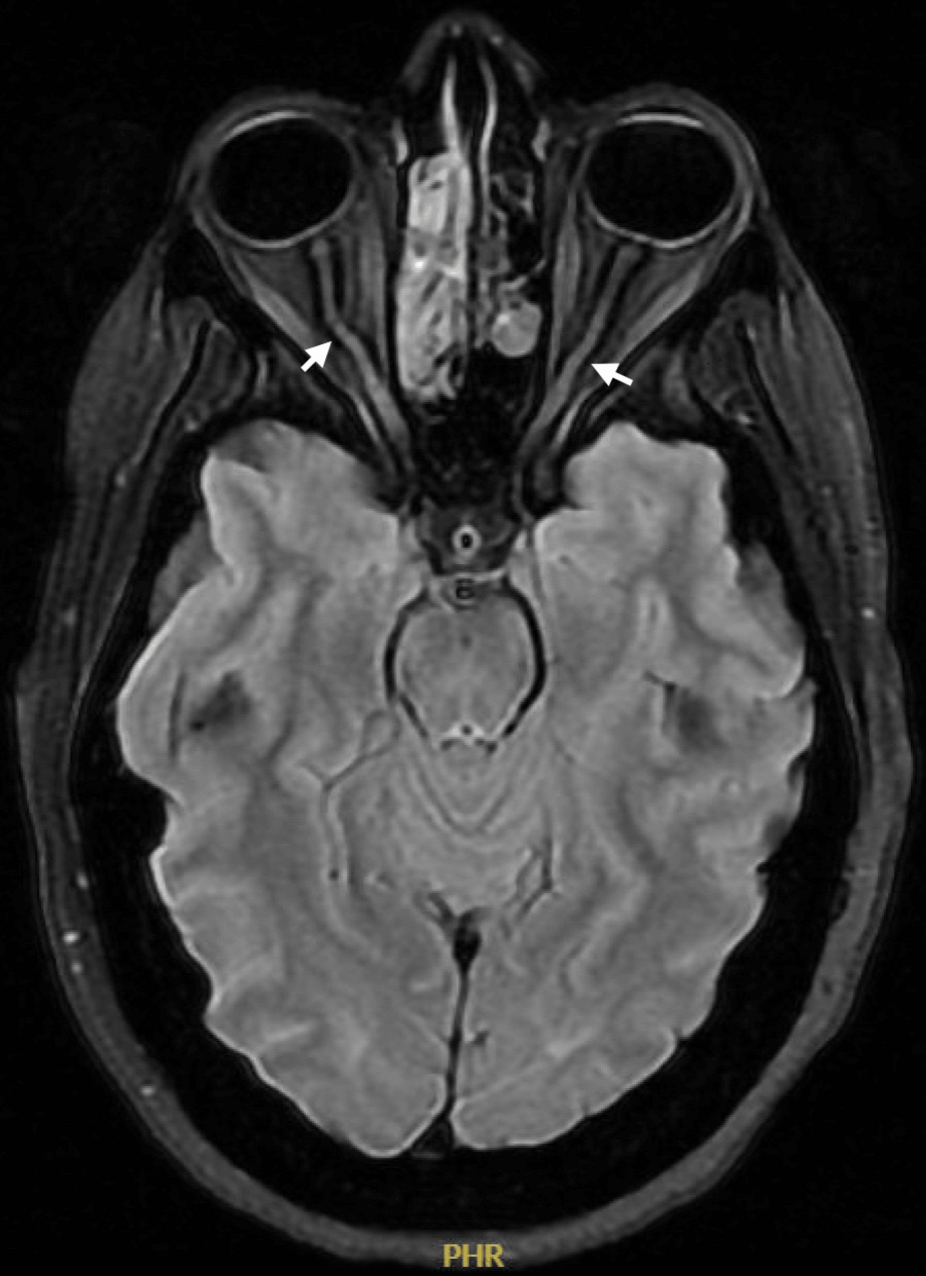
MRI (axial view) T2-weighted fat suppression showing bilateral tortuous optic nerve (arrows).

MRI scans confirmed a large anterior skull base defect with a meningoencephalocele containing the anterior cerebral arteries, along with diffuse calvarial thickening and narrowing of the skull base foramina. Signs indicative of increased intracranial pressure were found as bilateral tortuous optic nerves with enlarged surrounding CSF sleeves, partially empty sella turcica, and expanded Meckel's cave (Figure 2). Routine laboratory tests were all within normal ranges (Table 1). 

**Table 1:** Laboratory test results INR: international normalized ratio; MCV: mean corpuscular volume; MCH: mean corpuscular hemoglobin

Parameter	Patient Value	Reference Range
Potassium	3.41	3.5–5.0 mmol/L
Chloride	113.00 (H)	98–107 mmol/L
Anion Gap	9.61	8–12 mmol/L
Albumin	36.40	35–50 g/L
Calcium	2.17	2.2–2.6 mmol/L
Corrected Calcium	2.24	2.2–2.6 mmol/L
Phosphorus	1.02	0.8–1.5 mmol/L
INR	1.10	0.8–1.2
Auto WBC	9.64	4.0–11.0 x10^9^/L
Hemoglobin	9.8 (L)	12–16 g/dL
Hematocrit	31 (L)	37–47%
MCV	74.3 (L)	80–100 fL
MCH	23.8 (L)	27–32 pg
Platelets	299	150–400 x10^9^/L

The patient underwent a transnasal endoscopic skull base repair with intrathecal fluorescein injection, lumbar drain insertion, and facia late graft, jointly with Neurosurgery. Intraoperatively, it was seen that she had multiple base-of-skull defects: at the right fovea ethmoidalis extending into the right lateral lamella, there was a significant defect measuring 7x6 mm with a large encephalocele, a substantial defect at the right supraorbital ethmoid (SOE) measuring 54 mm with an encephalocele, and a linear defect at the right cribriform plate measuring 42 mm with a small meningocele. Also, a small defect at the left superior turbinate lamella was noted, measuring 4x4 mm with a small meningocele. Right large fovea and left lateral lamella defects were repaired with underlay fascial lata and overlay nasoseptal flaps. The right SOE defect was repaired with fat graft and overlay nasoseptal flap. The right small cribriform defect was repaired with fat graft and overlay mucosal graft that was taken from the middle turbinate.

The patient was discharged on the fifth postoperative day without any complications. We followed the patient every three months for the first year post surgery, then every six months to date and she has been doing well and has not evidenced further CSF leak. The patient was referred to the genetic department in our institute, and the subsequent evaluations showed a very rare mutation of *LRP4* gene. Mutation and clinical findings highly correlated with Cenani-Lenz syndactyly syndrome.

## Discussion

The differential diagnosis for cranial hyperostosis includes Paget's disease, acromegaly, osteopetrosis, long-term use of antiepileptic agents, and hyperparathyroidism, as well as the focal processes fibrous dysplasia, meningioma, ossifying fibroma, and HFI [7].

Although the literature on HCI remains sparse, HFI has significantly more documented cases, providing a clearer understanding of its clinical presentations and pathophysiology. Both conditions involve hyperossification, but HCI uniquely affects internal cranial surfaces and often shows a genetic basis, while HFI is predominantly associated with environmental and hormonal factors [8-10]. The lack of extensive studies on HCI underscores the need for further research to differentiate it fully from HFI and elucidate its unique characteristics.

This case illustrates the multifaceted nature of HCI, a very rare and not well-described entity with neurologic, genetic, and surgical management. Of note, our case is the first, since to the best of our knowledge, CSF leakage was not described in the literature earlier in HCI. Another documented case of HCI featured a patient presenting with dysphagia and bilateral vocal cord paralysis causing critical dyspnea [11]. Yet another documented case featured a patient with short-term memory loss [12]. These highlight the varied presentations of HCI. HCI can also present with subtle or nonspecific symptoms; thus, imaging studies are very important for diagnosis. The identification of the *LRP4* mutation in this patient establishes a possible genetic link between HCI and systemic syndromic conditions like Cenani-Lenz syndactyly syndrome.

In this case, surgical repair successfully treated the multiple defects of the skull base and stopped the leakage of CSF. This is a very good example of the value of a multidisciplinary team approach in dealing with complex cases: rhinology, neurosurgery, and genetics involved together. Moreover, the favorable postoperative outcomes of the patient underlined the efficacy of endoscopic techniques for skull base repair.

Even with this well-managed case, the pathophysiology of HCI still needs further research. More studies are required regarding the exact mechanism of the thickening of cranial bones, its association with raised intracranial pressure, and various genetic mutations. Long-term follow-up of such cases will be very important to understand the natural history of the condition and its systemic implications.

## Conclusions

This case was a complex one with diagnostic difficulty associated with HCI, presenting a constellation of symptoms and imaging features integral to diagnosis, pointing toward the importance of multidisciplinary collaboration in such cases. Surgical intervention was successful in addressing the defects at the skull base, and genetic testing revealed a rare mutation in the *LRP4* gene, thus linking the clinical findings to Cenani-Lenz syndactyly syndrome.

Such findings emphasize the importance of considering genetic and systemic associations in patients presenting with atypical cranial hyperostosis. Early detection and intervention can help avoid complications such as CSF leaks and treat potential systemic manifestations. Further studies are required to elucidate the exact pathophysiology, genetic basis, and long-term sequelae of HCI, thus leading to better management strategies and patient care.
